# Superconvergence of semidiscrete finite element methods for bilinear parabolic optimal control problems

**DOI:** 10.1186/s13660-017-1334-y

**Published:** 2017-03-16

**Authors:** Yuelong Tang, Yuchun Hua

**Affiliations:** 1 Institute of Computational Mathematics, College of Science, Hunan University of Science and Engineering, Yongzhou, Hunan 425100 China; 2 Office of Continuing Education, Peking University, Beijing, 100871 China

**Keywords:** 49J20, 65M60, superconvergence, finite element method, bilinear parabolic optimal control problems

## Abstract

In this paper, a semidiscrete finite element method for solving bilinear parabolic optimal control problems is considered. Firstly, we present a finite element approximation of the model problem. Secondly, we bring in some important intermediate variables and their error estimates. Thirdly, we derive a priori error estimates of the approximation scheme. Finally, we obtain the superconvergence between the semidiscrete finite element solutions and projections of the exact solutions. A numerical example is presented to verify our theoretical results.

## Introduction

We consider the following bilinear parabolic optimal control problem:

1.1 $$\begin{aligned} &\min _{u\in K}\frac{1}{2} \int_{0}^{T} \bigl(\bigl\| y(t,x)-y_{d}(t,x)\bigr\| _{L^{2}(\Omega)}^{2}+\bigl\| u(t,x)\bigr\| _{L^{2}(\Omega)}^{2} \bigr)\,dt, \end{aligned}$$

1.2 $$\begin{aligned} &\partial_{t} y(t,x)-\operatorname{div}\bigl(A(x)\nabla y(t,x) \bigr)+u(t,x)y(t,x)=f(t,x), \quad t\in J, x\in\Omega, \end{aligned}$$

1.3 $$\begin{aligned} &y(t,x)=0, \quad t\in J, x\in\partial\Omega, \end{aligned}$$

1.4 $$\begin{aligned} &y(0,x)=y_{0}(x), \quad x\in\Omega, \end{aligned}$$

where $\Omega\in\mathbb{R}^{2}$ is a convex polygon with the boundary *∂*Ω, and $J=[0,T]$ ($0< T<+\infty$). The coefficient matrix $A(x)=(a_{ij}(x))_{2\times2}\in [W^{1,\infty}(\bar{\Omega})]^{2\times2}$ is a symmetric and positive definite. Moreover, we assume that $f(t,x)\in C(J;L^{2}(\Omega))$, $y_{0}(x)\in H_{0}^{1}(\Omega )$, and the admissible control set *K* is defined by

$$K=\bigl\{ v(t,x)\in L^{2}\bigl(J;L^{2}(\Omega)\bigr): a\leq v(t,x)\leq b, \mbox{a.e. in } J\times\Omega \bigr\} , $$

where $0\leq a< b$ are real numbers.

There has been a wide range of research on finite element approximation of elliptic optimal control problems. For finite element solving linear and semilinear elliptic control problems, a priori error estimates were investigated in [1] and [2], and superconvergence were established in [3] and [4], respectively. Yang et al. [5] obtained the superconvergence of finite element approximation of bilinear elliptic control problems. In addition, some similar results of mixed finite element approximation for linear elliptic control problems can be found in [6, 7].

In recent years, there are a lot of related works on finite element approximation of parabolic optimal control problems, mostly focused on linear or semilinear cases. A priori error estimates of space-time finite element and standard finite element approximation for linear parabolic control problem were derived in [8] and [9]. The superconvergence of variational discretization and standard finite element approximation for semilinear parabolic control problem can be found in [10] and [11], respectively.

As far as we know, there has been little work done on bilinear parabolic control problems. In this paper, we purpose to obtain the superconvergence properties of semidiscrete finite element method for bilinear parabolic optimal control problems.

We adopt the notation $W^{m,q}(\Omega)$ for Sobolev spaces on Ω with norm $\|\cdot\|_{W^{m,q}(\Omega)}$ and seminorm $|\cdot|_{W^{m,q}(\Omega)}$. We set $H_{0}^{1}(\Omega)\equiv \{v \in H^{1}(\Omega): v|_{\partial\Omega} =0 \}$ and denote $W^{m,2}(\Omega)$ by $H^{m}(\Omega)$. We denote by $L^{s}(J;W^{m,q}(\Omega))$ the Banach space of all $L^{s}$ integrable functions from *J* into $W^{m,q}(\Omega)$ with norm $\|v\|_{L^{s}(J;W^{m,q}(\Omega))}=(\int_{0}^{T}\|v\|_{W^{m,q}(\Omega )}^{s}\,dt)^{\frac{1}{s}}$ for $s\in[1,\infty)$ and the standard modification for $s=\infty$. Similarly, we can define the space $H^{l}(J;W^{m,q}(\Omega))$ and $C^{k}(J;W^{m,q}(\Omega))$ (see e.g. [12]). In addition, let *c* or *C* be generic positive constants.

The rest of this paper is organized as follows. A semidiscrete finite element approximation of (1.1)-(1.4) is presented in Section 2. Some important intermediate variables and their error estimates are introduced in Section 3. In Section 4, a priori error estimates of the approximation scheme are derived. In Section 5, the superconvergence between projections of the exact solutions and the finite element solutions is obtained. A numerical example is presented to illustrate our theoretical results in the last section.

## A semidiscrete finite element approximation

We now consider a standard semidiscrete finite element approximation of (1.1)-(1.4). To ease the exposition, we denote $L^{p}(J;W^{m,q}(\Omega))$ and $\|\cdot\| _{L^{p}(J;W^{m,q}(\Omega))}$ by $L^{p}(W^{m,q})$ and ${\|\cdot\|} _{L^{p}(W^{m,q})}$ respectively. Let $W=H_{0}^{1}(\Omega)$ and $U=L^{2}(\Omega )$. Moreover, we denote $\|\cdot\|_{H^{m}(\Omega)}$ and $\|\cdot\|_{L^{2}(\Omega)}$ by $\|\cdot\|_{m}$ and $\|\cdot\|$, respectively. Let

$$\begin{aligned} \begin{aligned} &a(v,w)= \int_{\Omega}(A\nabla v)\cdot \nabla w, \quad \forall v, w\in W, \\ &(f_{1},f_{2})= \int_{\Omega}f_{1}\cdot f_{2}, \quad\forall f_{1}, f_{2}\in U. \end{aligned} \end{aligned}$$

From the assumptions on *A* we have

$$\begin{aligned} \begin{aligned} a(v,v)\geq c\|v\|_{1}^{2},\qquad \bigl|a(v,w)\bigr|\leq C\|v\|_{1}\|w\|_{1}, \quad\forall v, w\in W. \end{aligned} \end{aligned}$$

The weak formulation of (1.1)-(1.4) can be read as follows:

2.1 $$\begin{aligned} &\min_{u\in K}\frac{1}{2} \int_{0}^{T} \bigl(\|y-y_{d} \|^{2}+\|u\|^{2} \bigr)\,dt, \end{aligned}$$

2.2 $$\begin{aligned} &(\partial_{t} y,w)+a(y,w)+(u y,w)=(f,w),\quad \forall w\in W, t \in J, \end{aligned}$$

2.3 $$\begin{aligned} &y(x,0)=y_{0}(x),\quad \forall x\in\Omega. \end{aligned}$$

It follows from (see e.g. [13]) that problem (2.1)-(2.3) has at least one solution $(y,u)$ and that if the pair $(y,u)\in (H^{1}(L^{2})\cap L^{2}(H_{0}^{1}) )\times K$ is a solution of (2.1)-(2.3), then there is a costate $p\in (H^{1}(L^{2})\cap L^{2}(H_{0}^{1}) )$ such that the triplet $(y,p,u)$ meets the following optimality conditions:

2.4 $$\begin{aligned} &(\partial_{t} y,w)+a(y,w)+(u y,w)=(f,w), \quad\forall w\in W, t \in J, \end{aligned}$$

2.5 $$\begin{aligned} &y(0,x)=y_{0}(x), \quad\forall x\in\Omega, \end{aligned}$$

2.6 $$\begin{aligned} &{-}(\partial_{t} p,q)+a(q,p)+(u p,q)=(y-y_{d},q),\quad \forall q\in W, t\in J, \end{aligned}$$

2.7 $$\begin{aligned} &p(T,x)=0, \quad\forall x\in\Omega, \end{aligned}$$

2.8 $$\begin{aligned} & \int_{0}^{T}(u-y p,v-u)\,dt\geq0,\quad \forall v\in K. \end{aligned}$$

As in [3], it is easy to get the following lemma.

### Lemma 2.1

*Let* $(y,p,u)$ *be the solution of* (2.4)-(2.8). *Then*

2.9 $$\begin{aligned} u=\min \bigl(\max (a,y p ),b \bigr). \end{aligned}$$

Let $\mathbb{P}_{1}$ be the space of polynomials not exceeding 1, and $\mathcal{T}^{h}$ be regular triangulations of Ω such that $\bar{\Omega}=\bigcup _{\tau\in\mathcal{T}^{h}} \bar{\tau }$ and $h=\max _{\tau\in\mathcal{T}^{h}}\{h_{\tau}\}$, where $h_{\tau}$ denotes the diameter of the element *τ*. Furthermore, we set

$$\begin{aligned} \begin{aligned} &U_{h}= \bigl\{ v_{h}\in L^{2}(\Omega):v_{h}|_{\tau}=\text{constant}, \forall \tau \in\mathcal{T}^{h} \bigr\} , \\ &W_{h}= \bigl\{ v_{h}\in C(\bar{\Omega}):v_{h}|_{\tau} \in \mathbb{P}_{1}, \forall \tau\in\mathcal{T}^{h}, v_{h}|_{\partial\Omega}=0 \bigr\} . \end{aligned} \end{aligned}$$

As in [14], we assume that

$$K_{h}= \bigl\{ v_{h}\in U_{h}:a\leq v_{h}|_{\tau}\leq b, \forall \tau\in \mathcal{T}^{h} \bigr\} $$

is a closed convex set in $U_{h}$. We recast a semidiscrete finite element approximation of (2.1)-(2.3) as

2.10 $$\begin{aligned} &\min _{u_{h}\in L^{2}(K_{h})} \frac{1}{2} \int_{0}^{T} \bigl(\|y_{h}-y_{d} \|^{2}+\|u_{h}\|^{2} \bigr)\,dt, \end{aligned}$$

2.11 $$\begin{aligned} & (\partial_{t} y_{h},w_{h} )+a (y_{h},w_{h} )+(u_{h}y_{h},w_{h})= (f,w_{h} ), \quad\forall w_{h}\in W_{h}, t\in J, \end{aligned}$$

2.12 $$\begin{aligned} &y_{h}(0,x)=y_{0}^{h}(x), \quad \forall x\in \Omega, \end{aligned}$$

where $y_{0}^{h}(x)=R_{h}(y_{0}(x))$, and $R_{h}$ is an elliptic projection operator, which will be specified later.

It is well known that (2.10)-(2.12) again has a solution $(y_{h},u_{h})$ and that if the pair $(y_{h},u_{h})\in H^{1}(W_{h})\times L^{2}(K_{h})$ is a solution of (2.10)-(2.12), then there is a costate $p_{h}\in H^{1}(W_{h})$ such that the triplet $(y_{h},p_{h},u_{h})$ meets the following conditions:

2.13 $$\begin{aligned}& (\partial_{t} y_{h},w_{h})+a(y_{h},w_{h})+(u_{h} y_{h},w_{h})=(f,w_{h}), \quad \forall w_{h}\in W_{h}, t\in J, \end{aligned}$$

2.14 $$\begin{aligned}& y_{h}(0,x)=y^{h}_{0}(x),\quad \forall x\in \Omega, \end{aligned}$$

2.15 $$\begin{aligned}& {-}(\partial_{t} p_{h},q_{h})+a(q_{h},p_{h})+(u_{h} p_{h},q_{h})=(y_{h}-y_{d},q_{h}),\quad \forall q_{h}\in W_{h}, t\in J, \end{aligned}$$

2.16 $$\begin{aligned}& p_{h}(T,x)=0, \quad\forall x\in\Omega, \end{aligned}$$

2.17 $$\begin{aligned}& \int_{0}^{T}(u_{h}-y_{h} p_{h},v_{h}-u_{h})\,dt\geq0, \quad\forall v_{h}\in K_{h}. \end{aligned}$$

We introduce the averaging operator $\pi_{h}^{c}$ from *U* onto $U_{h}$ as

2.18 $$\begin{aligned} \bigl(\pi_{h}^{c} v\bigr)|_{\tau}= \frac{1}{|\tau|} \int_{\tau}v \,dx, \quad\forall \tau\in\mathcal{T}^{h}, \end{aligned}$$

where $|\tau|$ is the measure of *τ*. Then we can similarly derive the following lemma.

### Lemma 2.2

*Let* $(y_{h},p_{h},u_{h})$ *be the solution of* (2.13)-(2.17). *Then we have*

2.19 $$\begin{aligned} u_{h}=\min \bigl(\max \bigl(a,\pi_{h}^{c}(y_{h} p_{h}) \bigr),b \bigr). \end{aligned}$$

## Error estimates of intermediate variables

In this section, we introduce some important intermediate variables and derive some related error estimates. For all $v\in K$, let $y(v),p(v)\in H^{1}(L^{2})\cap L^{2}(H^{2})$ satisfy the following equations:

3.1 $$\begin{aligned}& \bigl(\partial_{t} y(v),w \bigr)+a \bigl(y(v),w \bigr)+\bigl(v y(v),w\bigr)= (f,w ), \quad\forall w\in W, t\in J, \end{aligned}$$

3.2 $$\begin{aligned}& y(v) (0,x)=y_{0}(x), \quad \forall x\in\Omega, \end{aligned}$$

3.3 $$\begin{aligned}& - \bigl(\partial_{t} p(v),q \bigr)+a \bigl(q,p(v) \bigr)+ \bigl(vp(v),q\bigr)= \bigl(y(v)-y_{d},q \bigr),\quad \forall q\in W, t\in J, \end{aligned}$$

3.4 $$\begin{aligned}& p(v) (T,x)=0, \quad \forall x\in\Omega. \end{aligned}$$

Let $y_{h}(v)$, $p_{h}(v)$ meet the following system:

3.5 $$\begin{aligned}& \bigl(\partial_{t} y_{h}(v),w_{h} \bigr)+a \bigl(y_{h}(v),w_{h} \bigr)+\bigl(v y_{h}(v),w_{h} \bigr)= (f,w_{h} ), \quad\forall w_{h}\in W_{h}, t \in J, \end{aligned}$$

3.6 $$\begin{aligned}& y_{h}(v) (0,x)=y_{0}^{h}(x), \quad \forall x \in\Omega, \end{aligned}$$

3.7 $$\begin{aligned}& - \bigl(\partial_{t} p_{h}(v),q_{h} \bigr)+a \bigl(q_{h},p_{h}(v) \bigr)+\bigl(v p_{h}(v),q_{h} \bigr)= \bigl(y_{h}(v)-y_{d},q_{h} \bigr), \quad\forall q_{h}\in W_{h}, t\in J, \end{aligned}$$

3.8 $$\begin{aligned}& p_{h}(v) (T,x)=0, \quad\quad\forall x\in\Omega. \end{aligned}$$

If $(y,p,u)$ and $(y_{h},p_{h},u_{h})$ are the solutions of (2.4)-(2.8) and (2.13)-(2.17), respectively, then $(y,p)=(y(u),p(u))$ and $(y_{h},p_{h})=(y_{h}(u_{h}),p_{h}(u_{h}))$.

We define an elliptic projection operator $R_{h}:W\rightarrow W_{h}$ that satisfies

3.9 $$\begin{aligned} a(R_{h}\phi-\phi,w_{h})=0, \quad\forall \phi\in W,w_{h}\in W_{h}, \end{aligned}$$

and the $L^{2}$-orthogonal projection operator $Q_{h}:U\rightarrow U_{h}$ that satisfies

3.10 $$\begin{aligned} (Q_{h} \psi-\psi,v_{h})=0, \quad\forall \psi\in U,v_{h}\in U_{h}. \end{aligned}$$

They have the following properties (see e.g. [4]):

3.11 $$\begin{aligned} &\|R_{h} \phi-\phi\|_{s}\leq Ch^{2-s}\|\phi \|_{2}, \quad\forall \phi \in H^{2}(\Omega),s=0,1, \end{aligned}$$

3.12 $$\begin{aligned} &\|Q_{h}\psi-\psi\|_{-s}\leq Ch^{1+s}| \psi|_{1}, \quad\forall \psi \in H^{1}(\Omega),s=0,1. \end{aligned}$$

The following lemmas are very important for a priori error estimates and superconvergence analysis.

### Lemma 3.1

*For any* $v\in K$, *if there exists a constant* $c>0$ *such that*

3.13 $$\begin{aligned} c\|w\|_{1}^{2}\leq a(w,w)+(v w,w), \quad\forall w\in W, \end{aligned}$$

*then* (3.1)-(3.4) *and* (3.5)-(3.8) *have unique solutions*, *respectively*. *Assuming that* $y(v),p(v)\in H^{1}(H^{2})$, *we have*

3.14 $$\begin{aligned}& \bigl\| y(v)-y_{h}(v)\bigr\| _{L^{\infty}(L^{2})}+\bigr\| y(v)-y_{h}(v) \bigr\| _{L^{2}(H^{1})}\leq C h, \end{aligned}$$

3.15 $$\begin{aligned}& \bigr\| p(v)-p_{h}(v)\bigr\| _{L^{\infty}(L^{2})}+\bigr\| p(v)-p_{h}(v) \bigr\| _{L^{2}(H^{1})}\leq C h. \end{aligned}$$

### Proof

It follows from (3.1)-(3.4) and (3.5)-(3.8) that

3.16 $$\begin{aligned}& \begin{aligned}[b]& \bigl(\partial_{t} \bigl(y(v)-y_{h}(v)\bigr),w_{h} \bigr)+a \bigl(y(v)-y_{h}(v),w_{h} \bigr)+\bigl(v \bigl(y(v)-y_{h}(v)\bigr),w_{h}\bigr)=0, \\ &\quad\forall w_{h}\in W_{h}, t\in J, \end{aligned} \end{aligned}$$

3.17 $$\begin{aligned}& y(v) (0,x)-y_{h}(v) (0,x)=y_{0}(x)-y_{0}^{h}(x), \quad\forall x\in\Omega , \end{aligned}$$

3.18 $$\begin{aligned}& \begin{aligned}[b]&{-} \bigl(\partial_{t} \bigl(p(v)-p_{h}(v)\bigr),q_{h} \bigr)+a \bigl(q_{h},p(v)-p_{h}(v) \bigr)+\bigl(v \bigl(p(v)-p_{h}(v)\bigr),q_{h}\bigr) \\ &\quad= \bigl(y(v)-y_{h}(v),q_{h} \bigr), \quad\forall q_{h}\in W_{h}, t\in J, \end{aligned} \end{aligned}$$

3.19 $$\begin{aligned}& p(v) (T,x)-p_{h}(v) (T,x)=0, \quad\forall x\in \Omega, \end{aligned}$$

Letting $w_{h}=R_{h}y(v)-y_{h}(v)$ in (3.16), we obtain, for any $t\in J$,

3.20 $$\begin{aligned} \begin{aligned}[b]0={}& \bigl(\partial_{t} \bigl(y(v)-y_{h}(v)\bigr),R_{h} y(v)-y_{h}(v) \bigr)+a \bigl(y(v)-y_{h}(v),R_{h} y(v)-y_{h}(v) \bigr) \\ &+\bigl(v \bigl(y(v)-y_{h}(v)\bigr),R_{h} y(v)-y_{h}(v)\bigr). \end{aligned} \end{aligned}$$

Applying (3.13) to (3.20), we have

3.21 $$\begin{aligned} \begin{aligned}[b] &\frac{1}{2}\frac{d}{dt} \bigl(\bigr\| y(v)-y_{h}(v)\bigr\| ^{2}\bigr)+c\bigr\| y(v)-y_{h}(v) \bigr\| _{1}^{2} \\ &\quad\leq \bigl(\partial_{t} \bigl(y(v)-y_{h}(v) \bigr),y(v)-y_{h}(v) \bigr)+a \bigl(y(v)-y_{h}(v),y(v)-y_{h}(v) \bigr) \\ &\qquad+\bigl(v \bigl(y(v)-y_{h}(v)\bigr),y(v)-y_{h}(v)\bigr) \\ &\quad= \bigl(\partial_{t} \bigl(y(v)-y_{h}(v) \bigr),y(v)-R_{h} y(v) \bigr)+a \bigl(y(v)-y_{h}(v),y(v)-R_{h} y(v) \bigr) \\ &\qquad+\bigl(v \bigl(y(v)-y_{h}(v)\bigr),y(v)-R_{h} y(v)\bigr). \end{aligned} \end{aligned}$$

From (3.11) and (3.21), Hölder’s inequality, Young’s inequality with *ϵ*, and Gronwall’s inequality, we derive (3.14). Similarly, we can get (3.15). □

### Lemma 3.2

*For any* $\upsilon, \omega\in K$, *let* $(y(\upsilon),p(\upsilon))$ *and* $(y(\omega),p(\omega))$ *be the solutions of* (3.1)-(3.4), $(y_{h}(\upsilon),p_{h}(\upsilon))$, *and let* $(y_{h}(\omega ),p_{h}(\omega))$ *be the solutions of* (3.5)-(3.8). *Then*

3.22 $$\begin{aligned}& \big\| y(\upsilon)-y(\omega)\big\| _{L^{\infty}(L^{2})}+\big\| y(\upsilon)-y(\omega ) \big\| _{L^{2}(H^{1})}\leq C\|\upsilon-\omega\|_{L^{2}(H^{-1})}, \end{aligned}$$

3.23 $$\begin{aligned}& \big\| p(\upsilon)-p(\omega)\big\| _{L^{\infty}(L^{2})}+\big\| p(\upsilon)-p(\omega ) \big\| _{L^{2}(H^{1})}\leq C\|\upsilon-\omega\|_{L^{2}(H^{-1})}, \end{aligned}$$

3.24 $$\begin{aligned}& \big\| y_{h}(\upsilon)-y_{h}(\omega)\big\| _{L^{\infty}(L^{2})}+ \big\| y_{h}(\upsilon )-y_{h}(\omega)\big\| _{L^{2}(H^{1})}\leq C\| \upsilon-\omega\| _{L^{2}(H^{-1})}, \end{aligned}$$

3.25 $$\begin{aligned}& \big\| p_{h}(\upsilon)-p_{h}(\omega)\big\| _{L^{\infty}(L^{2})}+ \big\| p_{h}(\upsilon )-p_{h}(\omega)\big\| _{L^{2}(H^{1})}\leq C\| \upsilon-\omega\| _{L^{2}(H^{-1})}. \end{aligned}$$

### Proof

For any $\upsilon,\omega\in K$, it is clear that

3.26 $$\begin{aligned}& \begin{aligned}[b]&\bigl(\partial_{t} \bigl(y(\upsilon)-y(\omega)\bigr),w\bigr)+a\bigl(y(\upsilon )-y(\omega),w\bigr)+ \bigl(\upsilon\bigl(y(\upsilon)-y(\omega)\bigr),w\bigr) \\ &\quad=\bigl(\omega-\upsilon,wy(\omega)\bigr), \quad\forall w\in W,t\in J, \end{aligned} \end{aligned}$$

3.27 $$\begin{aligned}& y(\upsilon) (0,x)-y(\omega) (0,x)=0, \quad\forall x\in\Omega , \end{aligned}$$

3.28 $$\begin{aligned}& \begin{aligned}[b]&{-}\bigl(\partial_{t} \bigl(p(\upsilon)-p(\omega)\bigr),q\bigr)+a\bigl(p(\upsilon )-p(\omega),q\bigr)+ \bigl(\upsilon\bigl(p(\upsilon)-p(\omega)\bigr),q\bigr) \\ &\quad=\bigl(y(\upsilon)-y(\omega),q\bigr)+\bigl(\omega-\upsilon,q p(\omega)\bigr), \quad\forall q\in W,t\in J, \end{aligned} \end{aligned}$$

3.29 $$\begin{aligned}& p(\upsilon) (T,x)-p(\omega) (T,x)=0, \quad\forall x\in\Omega . \end{aligned}$$

Inequalities (3.22) and (3.23) follow from the regularity estimates of (3.26)-(3.27) and (3.28)-(3.29), respectively. Analogously, we can derive (3.24) and (3.25). □

### Lemma 3.3

*Let* $(y,p,u)$ *be the solution of* (2.4)-(2.8). *Assume that* $u\in L^{2}(H^{1})$. *We have*

3.30 $$\begin{aligned}& \big\| y_{h}(Q_{h}u)-y_{h}(u)\big\| _{L^{\infty}(L^{2})}+ \big\| y_{h}(Q_{h}u)-y_{h}(u)\big\| _{L^{2}(H^{1})}\leq Ch^{2}, \end{aligned}$$

3.31 $$\begin{aligned}& \big\| p_{h}(Q_{h}u)-p_{h}(u)\big\| _{L^{\infty}(L^{2})}+ \big\| p_{h}(Q_{h}u)-p_{h}(u)\big\| _{L^{2}(H^{1})}\leq Ch^{2}. \end{aligned}$$

### Proof

It follows from (3.12) that

3.32 $$\begin{aligned} \|Q_{h}u-u\|_{L^{2}(H^{-1})}\leq Ch^{2}\|u \|_{L^{2}(H^{1})}. \end{aligned}$$

Setting $\upsilon=Q_{h}u$ and $\omega=u$ in (3.24)-(3.25), we obtain (3.30)-(3.31). □

### Lemma 3.4

*Let* $(y(v),p(v))$ *be the solution of* (3.5)-(3.8) *with* $v\in K$. *Suppose that* $y(v),p(v)\in H^{1}(H^{2})$. *Then the following estimates hold*:

3.33 $$\begin{aligned}& \big\| R_{h}y(v)-y_{h}(v)\big\| _{L^{\infty}(L^{2})}+ \big\| R_{h}y(v)-y_{h}(v)\big\| _{L^{2}(H^{1})}\leq Ch^{2}, \end{aligned}$$

3.34 $$\begin{aligned}& \big\| R_{h}p(v)-p_{h}(v)\big\| _{L^{\infty}(L^{2})}+ \big\| R_{h}p(v)-p_{h}(v)\big\| _{L^{2}(H^{1})}\leq Ch^{2}. \end{aligned}$$

### Proof

It follows from the definition of $R_{h}$ and (3.1)-(3.8) that

3.35 $$\begin{aligned}& \begin{aligned}[b]& \bigl(\partial_{t} \bigl(y(v)-y_{h}(v)\bigr),w_{h} \bigr)+a \bigl(R_{h}y(v)-y_{h}(v),w_{h} \bigr)+\bigl(v \bigl(y(v)-y_{h}(v)\bigr),w_{h}\bigr)=0, \\ &\quad\forall w_{h}\in W_{h}, t\in J, \end{aligned} \end{aligned}$$

3.36 $$\begin{aligned}& y(v) (0,x)-y_{h}(v) (0,x)=y_{0}(x)-y_{0}^{h}(x), \quad\forall x\in\Omega , \end{aligned}$$

3.37 $$\begin{aligned}& \begin{aligned}[b]&{-} \bigl(\partial_{t} \bigl(p(v)-p_{h}(v)\bigr),q_{h} \bigr)+a \bigl(q_{h},R_{h}p(v)-p_{h}(v) \bigr)+\bigl(v \bigl(p(v)-p_{h}(v)\bigr),q_{h}\bigr) \\ &\quad= \bigl(y(v)-y_{h}(v),q_{h} \bigr), \quad\forall q_{h}\in W_{h}, t\in J, \end{aligned} \end{aligned}$$

3.38 $$\begin{aligned}& p(v) (T,x)-p(v)_{h}(T,x)=0, \quad\forall x\in \Omega. \end{aligned}$$

Let $w_{h}=R_{h}y(v)-y_{h}(v)$ in (3.35). From Hölder’s inequality, Young’s inequality with *ϵ*, and (3.11) we derive

3.39 $$\begin{aligned} \begin{aligned} [b]&\frac{1}{2}\frac{d}{dt} \bigl(\big\| R_{h}y(v)-y_{h}(v)\big\| ^{2}\bigr)+c \big\| R_{h}y(v)-y_{h}(v)\big\| _{1}^{2} \\ &\quad\leq \bigl(\partial_{t} \bigl(R_{h}y(v)-y_{h}(v) \bigr),R_{h}y(v)-y_{h}(v) \bigr)+a \bigl(R_{h}y(v)-y_{h}(v),R_{h}y(v)-y_{h}(v) \bigr) \\ &\qquad+\bigl(v \bigl(R_{h}y(v)-y_{h}(v)\bigr),R_{h}y(v)-y_{h}(v) \bigr) \\ &\quad= \bigl(\partial_{t} \bigl(R_{h}y(v)-y(v) \bigr),R_{h} y(v)-y_{h}(v) \bigr)+\bigl(v \bigl(R_{h}y(v)-y(v)\bigr),R_{h} y(v)-y_{h}(v)\bigr) \\ &\quad\leq C(\epsilon)h^{2}\big\| \partial_{t} y(v) \big\| _{2}^{2}+C(\epsilon)h^{2}\big\| y(v)\big\| _{2}^{2}+2\epsilon\big\| R_{h}y(v)-y_{h}(v) \big\| ^{2}. \end{aligned} \end{aligned}$$

Note that

3.40 $$\begin{aligned} R_{h}y(v) (0,x)-y_{h}(v) (0,x)=0. \end{aligned}$$

Estimate (3.33) follows from (3.39) and Gronwall’s inequality. Similarly, we can obtain (3.34). □

## A priori error estimates

In this section, we derive a priori error estimates of the approximation scheme (2.13)-(2.17). For ease of exposition, we set

$$\begin{aligned} \begin{aligned} &J(u)= \int_{0}^{T} \bigl(\|y-y_{d} \|^{2}+\|u\|^{2} \bigr)\,dt, \\ &J_{h}(u_{h})= \int_{0}^{T} \bigl(\|y_{h}-y_{d} \|^{2}+\|u_{h}\|^{2} \bigr)\,dt. \end{aligned} \end{aligned}$$

It is easy to show that

$$\begin{aligned}& \bigl(J^{\prime}(u),v\bigr)= \int_{0}^{T}(u-y p,v)\,dt, \\& \bigl(J_{h}^{\prime}(u_{h}),v\bigr)= \int_{0}^{T}(u_{h}-y_{h}p_{h},v)\,dt. \end{aligned}$$

As in [15], we assume that there exist neighborhoods of the exact solution *u* or of the approximation solution $u_{h}$ in *K* and a constant $c_{0}>0$ such that, for any *v* or $v_{h}$ in this neighborhood, the objective functional satisfies the following convexity conditions:

4.1 $$\begin{aligned}& c_{0}\|v-u\|_{L^{2}(L^{2})}^{2}\leq\bigl(J^{\prime}(v)-J^{\prime}(u),v-u \bigr), \end{aligned}$$

4.2 $$\begin{aligned}& c_{0}\|v_{h}-u_{h}\|_{L^{2}(L^{2})}^{2} \leq\bigl(J_{h}^{\prime}(v_{h})-J_{h}^{\prime}(u_{h}),v_{h}-u_{h}\bigr). \end{aligned}$$

### Theorem 4.1

*Let* $(y,p,u)$ *and* $(y_{h},p_{h},u_{h})$ *be the solutions of* (2.4)-(2.8) *and* (2.13)-(2.17). *Suppose that* $y,p\in H^{1}(L^{2})\cap L^{2}(H^{1})$. *Then*

4.3 $$\begin{aligned}& \|u-u_{h}\|_{L^{2}(L^{2})}\leq C h, \end{aligned}$$

4.4 $$\begin{aligned}& \|y-y_{h}\|_{L^{\infty}(L^{2})}+\|y-y_{h}\|_{L^{2}(H^{1})}\leq C h, \end{aligned}$$

4.5 $$\begin{aligned}& \|p-p_{h}\|_{L^{\infty}(L^{2})}+\|p-p_{h}\|_{L^{2}(H^{1})}\leq C h. \end{aligned}$$

### Proof

It follows from (2.8), (2.17), (3.10), and (4.1) that

4.6 $$\begin{aligned} \begin{aligned}[b]&c_{0}\|u-u_{h} \|_{L^{2}(L^{2})}^{2} \\ &\quad\leq \bigl(J^{\prime}(u)-J^{\prime}(u_{h}),u-u_{h} \bigr) \\ &\quad= \int_{0}^{T}(u-y p,u-u_{h})\,dt- \int_{0}^{T}\bigl(u_{h}-y(u_{h})p(u_{h}),u-u_{h} \bigr)\,dt \\ &\quad\leq \int_{0}^{T}\bigl[(y p,u-u_{h})+(y_{h}p_{h},u_{h}-u)\\ &\qquad+(u_{h}-y_{h}p_{h},Q_{h} u-u)-\bigl(y p-y(u_{h})p(u_{h}),u-u_{h}\bigr) \bigr]\,dt \\ &\quad= \int_{0}^{T}\bigl[(u_{h}-y_{h}p_{h},Q_{h} u-u)+\bigl(y_{h}p_{h}-y(u_{h})p(u_{h}),u_{h}-u \bigr)\bigr]\,dt \\ &\quad= \int_{0}^{T}\bigl(y p-y(u_{h})p(u_{h}),Q_{h} u-u\bigr)\,dt+ \int _{0}^{T}\bigl(y(u_{h})p(u_{h})-y_{h}p_{h},Q_{h} u-u\bigr)\,dt \\ &\qquad+ \int_{0}^{T}(y p,u-Q_{h} u)\,dt+ \int_{0}^{T}\bigl(y_{h}p_{h}-y(u_{h})p(u_{h}),u_{h}-u \bigr)\,dt \\ &\quad:=I_{1}+I_{2}+I_{3}+I_{4}. \end{aligned} \end{aligned}$$

For the first term $I_{1}$, by the embedding inequality $\|v\| _{L^{4}(\Omega)}\leq C\|v\|_{H^{1}(\Omega)}$ and Young’s inequality with *ϵ* we get

4.7 $$\begin{aligned} \begin{aligned} [b]I_{1}={}& \int_{0}^{T}\bigl(y p-y(u_{h})p(u_{h}),Q_{h} u-u\bigr)\,dt \\ \leq{}&C(\epsilon)\|Q_{h} u-u\|_{L^{2}(L^{2})}^{2}+\epsilon C \big\| y(u)-y(u_{h})\big\| _{L^{2}(H^{1})}^{2}+\epsilon C \big\| p(u)-p(u_{h})\big\| _{L^{2}(H^{1})}^{2}. \end{aligned} \end{aligned}$$

By Hölder’s inequality and the embedding inequality $\|v\| _{L^{4}(\Omega)}\leq C\|v\|_{H^{1}(\Omega)}$ we have

4.8 $$\begin{aligned} \begin{aligned}[b] I_{2}={}& \int_{0}^{T}\bigl(y(u_{h})p(u_{h})-y_{h}p_{h},Q_{h} u-u\bigr)\,dt \\ \leq{}&C \bigl(\|Q_{h} u-u\|_{L^{2}(L^{2})}^{2}+ \big\| y_{h}-y(u_{h})\big\| _{L^{2}(H^{1})}^{2}+\big\| p_{h}-p(u_{h})\big\| _{L^{2}(H^{1})}^{2} \bigr) , \end{aligned} \end{aligned}$$

and the third term $I_{3}$ can be estimates as follows:

4.9 $$\begin{aligned} \begin{aligned}[b] I_{3}={}& \int_{0}^{T}(y p,u-Q_{h} u)\,dt \\ ={}& \int_{0}^{T}(y p-Q_{h} y Q_{h} p,u-Q_{h} u)\,dt \\ \leq{}&C \bigl(\|y-Q_{h} y\|_{L^{2}(H^{1})}^{2}+ \|p-Q_{h} p\|_{L^{2}(H^{1})}^{2}+\|Q_{h} u-u \|_{L^{2}(L^{2})}^{2} \bigr). \end{aligned} \end{aligned}$$

Applying Hölder’s inequality, the embedding inequality $\|v\| _{L^{4}(\Omega)}\leq C\|v\|_{H^{1}(\Omega)}$, and Young’s inequality with *ϵ* to $I_{4}$, we have

4.10 $$\begin{aligned} \begin{aligned}[b] I_{4}={}& \int_{0}^{T}\bigl(y_{h}p_{h}-y(u_{h})p(u_{h}),u_{h}-u \bigr)\,dt \\ \leq{}&C(\epsilon) \bigl(\big\| y_{h}-y(u_{h})\big\| _{L^{2}(H^{1})}^{2}+ \big\| p_{h}-p(u_{h})\big\| _{L^{2}(H^{1})}^{2} \bigr)+ \epsilon\|u-u_{h}\|_{L^{2}(L^{2})}^{2}. \end{aligned} \end{aligned}$$

According to (3.12), Lemmas 3.1-3.2, and (4.6)-(4.10), we obtain

4.11 $$\begin{aligned} \|u-u_{h}\|_{L^{2}(L^{2})}\leq C h. \end{aligned}$$

From (2.4)-(2.5) and (2.13)-(2.14) we have

4.12 $$\begin{aligned}& \bigl(\partial_{t} (y-y_{h}),w_{h} \bigr)+a(y-y_{h},w_{h})+(u y-u_{h} y_{h},w_{h})=0, \quad\forall w_{h}\in W_{h},t\in J, \end{aligned}$$

4.13 $$\begin{aligned}& y(0,x)-y_{h}(0,x)=y_{0}(x)-R_{h} y_{0}(x), \quad\forall x\in\Omega. \end{aligned}$$

Letting $w_{h}=R_{h}y-y_{h}$ in (4.12), we get, for any $t\in J$,

4.14 $$\begin{aligned} \begin{aligned}[b] &\bigl(\partial_{t} (y-y_{h}),y-y_{h}\bigr)+a(y-y_{h},y-y_{h})+ \bigl(u(y-y_{h}),y-y_{h}\bigr) \\ &\quad=\bigl(\partial_{t} (y-y_{h}),y-R_{h} y \bigr)+a(y-y_{h},y-R_{h} y)+\bigl(y_{h}(u_{h}-u),y-y_{h} \bigr) \\ &\qquad+\bigl(u(y-y_{h}),y-R_{h} y\bigr)+\bigl(y_{h}(u-u_{h}),y-R_{h} y\bigr). \end{aligned} \end{aligned}$$

From (3.11), (4.3), (4.14), Young’s inequality with *ϵ*, and Gronwall’s inequality we derive (4.4). It is paralleled to get (4.5). □

## Superconvergence analysis

In this section, we derive the superconvergence between projections of the exact solutions and approximation solutions. Let *u* be the solutions of (2.4)-(2.8). For a fixed $t^{*}$ ($0\leq {t^{*}\leq T}$), we divide Ω into the following subsets:

$$\begin{aligned} \begin{aligned} &\Omega^{+}= \Bigl\{ \bigcup\tau : \tau\subset\Omega, a< u\bigl(t^{*},\cdot\bigr)< b \Bigr\} , \\ &\Omega^{0}= \Bigl\{ \bigcup\tau : \tau\subset\Omega, u\bigl(t^{*},\cdot \bigr)\big|_{\tau}=a \mbox{ or } u\bigl(t^{*},\cdot \bigr)\big|_{\tau}=b \Bigr\} , \\ &\Omega^{-}=\Omega\setminus \bigl(\Omega^{+}\cup\Omega^{0} \bigr). \end{aligned} \end{aligned}$$

It is easy to see that these three subsets do not intersect with each other and $\Omega=\bar{\Omega}^{+}\cup\bar{\Omega}^{0}\cup \bar{\Omega}^{-}$. We assume that *u* and $\mathcal{T}_{h}$ are regular such that $\operatorname{meas} (\Omega^{-} )\leq Ch$ (see, e.g., [8]).

### Theorem 5.1

*Let* $(y,p,u)$ *and* $(y_{h},p_{h},u_{h})$ *be the solutions of* (2.4)-(2.8) *and* (2.13)-(2.17), *respectively*. *Assume that all the conditions in Lemmas* 3.1-3.4 *are valid and* $y,p\in L^{2}(L^{\infty})$. *Moreover*, *we suppose that the exact control*, *state*, *and costate solutions satisfy*

$$u, u-yp\in L^{2}\bigl(W^{1,\infty}\bigr). $$

*Then*, *we have*

5.1 $$\begin{aligned} \|Q_{h}u-u_{h}\|_{L^{2}(L^{2})}\leq Ch^{\frac{3}{2}}. \end{aligned}$$

### Proof

Letting $v_{h}=Q_{h}u$ in (2.17), we obtain the inequality

5.2 $$\begin{aligned} \int_{0}^{T}(u_{h}-y_{h}p_{h},Q_{h}u-u_{h})\,dt \geq0. \end{aligned}$$

It follows from the definition of $Q_{h}$, (4.2), and (5.1) that

5.3 $$\begin{aligned} \begin{aligned}[b] &c_{0}\|Q_{h}u-u_{h} \|_{L^{2}(L^{2})}^{2} \\ &\quad\leq \bigl(J_{h}^{\prime}(Q_{h}u)-J_{h}^{\prime}(u_{h}),Q_{h}u-u_{h} \bigr) \\ &\quad= \int_{0}^{T} \bigl(Q_{h}u-y_{h}(Q_{h}u)p_{h}(Q_{h}u),Q_{h}u-u_{h} \bigr)\,dt- \int _{0}^{T} (u_{h}-y_{h}p_{h},Q_{h}u-u_{h} )\,dt \\ &\quad\leq \int_{0}^{T} \bigl(u-y_{h}(Q_{h}u)p_{h}(Q_{h}u),Q_{h}u-u_{h} \bigr)\,dt \\ &\quad= \int_{0}^{T} (u-yp,Q_{h}u-u_{h} )\,dt+ \int_{0}^{T} \bigl(y_{h}(u)p_{h}(u)-y_{h}(Q_{h}u)p_{h}(Q_{h}u),Q_{h}u-u_{h} \bigr)\,dt \\ &\qquad+ \int_{0}^{T} \bigl(R_{h}yR_{h}p-y_{h}(u)p_{h}(u),Q_{h}u-u_{h} \bigr)\,dt+ \int _{0}^{T} (yp-R_{h}yR_{h}p,Q_{h}u-u_{h} )\,dt \\ &\quad=:I_{1}+I_{2}+I_{3}+I_{4}. \end{aligned} \end{aligned}$$

For the first term, at time $t^{*}$ ($0\leq t^{*}\leq T$), we have

5.4 $$\begin{aligned} (u-yp,Q_{h}u-u_{h} )= \biggl( \int_{\Omega^{+}}+ \int_{\Omega ^{0}}+ \int_{\Omega^{-}} \biggr) (u-yp ) (Q_{h}u-u_{h} )\,dx \end{aligned}$$

and

$$\begin{aligned} (Q_{h}u-u )|_{\Omega^{0}}=0. \end{aligned}$$

From (2.9) we get

$$\begin{aligned} (u-yp )|_{\Omega^{+}}=0. \end{aligned}$$

Hence,

5.5 $$\begin{aligned} \begin{aligned}[b] I_{1}={}& \int_{0}^{T} \int_{\Omega^{-}} (u-yp ) (Q_{h}u-u_{h} )\,dx\,dt \\ ={}& \int_{0}^{T} \int_{\Omega^{-}} \bigl( (u-yp )-Q_{h} (u-yp ) \bigr) (Q_{h}u-u_{h} )\,dx\,dt \\ \leq{}&Ch^{2} \int_{0}^{T}\|u-yp\|_{1,\Omega^{-}}\|u \|_{1,\Omega^{-}}\,dt \\ \leq{}&Ch^{2} \int_{0}^{T}\|u-yp\|_{W^{1,\infty}}\|u \|_{W^{1,\infty}}\cdot \operatorname{meas}\bigl(\Omega^{-}\bigr)\,dt \\ \leq{}&Ch^{3} \bigl(\|u-yp\|_{L^{2}(W^{1,\infty})}^{2}+\|u \|_{L^{2}(W^{1,\infty })}^{2} \bigr). \end{aligned} \end{aligned}$$

By using Hölder’s inequalty, the embedding inequality $\|v\| _{L^{4}(\Omega)}\leq C\|v\|_{H^{1}(\Omega)}$, and Young’s inequality, $I_{2}$ and $I_{3}$ can be estimated as follows:

5.6 $$\begin{aligned} \begin{aligned}[b] I_{2}\leq{}& C(\epsilon) \bigl(\big\| y_{h}(u)-y_{h}(Q_{h}u) \big\| _{L^{2}(H^{1})}^{2}+\big\| p_{h}(u)-p_{h}(Q_{h}u) \big\| _{L^{2}(H^{1})}^{2} \bigr)\\ &+\epsilon\|Q_{h}u-u_{h} \|_{L^{2}(L^{2})}^{2} \end{aligned} \end{aligned}$$

and

5.7 $$\begin{aligned} I_{3}\leq C(\epsilon) \bigl(\big\| R_{h}y-y_{h}(u) \big\| _{L^{2}(H^{1})}^{2}+\big\| R_{h}p-p_{h}(u)\big\| _{L^{2}(H^{1})}^{2} \bigr)+\epsilon\|Q_{h}u-u_{h} \|_{L^{2}(L^{2})}^{2}. \end{aligned}$$

In addition, noting that $y,p\in L^{2}(L^{\infty})$, we have

5.8 $$\begin{aligned} I_{4}\leq C(\epsilon) \bigl(\|R_{h}y-y \|_{L^{2}(L^{2})}^{2}+\|R_{h}p-p\| _{L^{2}(L^{2})}^{2} \bigr)+\epsilon\|Q_{h}u-u_{h}\|_{L^{2}(L^{2})}^{2}. \end{aligned}$$

It follows from (5.3)-(5.8), (3.11), and Lemmas 3.3-3.4 that (5.1) holds for small enough *ϵ*. □

### Theorem 5.2

*Let* $(y,p,u)$ *and* $(y_{h},p_{h},u_{h})$ *be the solutions of* (2.4)-(2.8) *and* (2.13)-(2.17), *respectively*. *Assume that all the conditions in Theorem* 5.1 *are valid*. *Then*

5.9 $$\begin{aligned}& \|y_{h}-R_{h}y\|_{L^{\infty}(L^{2})}+\|y_{h}-R_{h}y \|_{L^{2}(H^{1})}\leq Ch^{\frac {3}{2}}, \end{aligned}$$

5.10 $$\begin{aligned}& \|p_{h}-R_{h}p\|_{L^{\infty}(L^{2})}+\|p_{h}-R_{h}p \|_{L^{2}(H^{1})}\leq Ch^{\frac {3}{2}}. \end{aligned}$$

### Proof

From the definition of $R_{h}$, (2.4)-(2.7), and (2.13)-(2.16), for any $w_{h} \mbox{ or } q_{h}\in W_{h}$ and $t\in J$, we have

5.11 $$\begin{aligned}& \begin{aligned}[b]&\bigl(\partial_{t} (y_{h}-R_{h}y ),w_{h}\bigr)+a(y_{h}-R_{h}y,w_{h})+ \bigl(u (y_{h}-R_{h}y ),w_{h}\bigr) \\ &\quad=\bigl(\partial_{t} (y-R_{h}y ),w_{h}\bigr)+ \bigl(u(y-R_{h}y),w_{h}\bigr)\\ &\qquad+\bigl(y_{h}(u-Q_{h}u),w_{h} \bigr)+\bigl(y_{h}(Q_{h}u-u_{h}),w_{h} \bigr), \end{aligned} \end{aligned}$$

5.12 $$\begin{aligned}& y_{h}(0,x)-R_{h}y(0,x)=0, \quad\forall x\in \Omega, \end{aligned}$$

5.13 $$\begin{aligned}& \begin{aligned}[b]&{-}\bigl(\partial_{t} (p_{h}-R_{h}p ),q_{h}\bigr)+a(q_{h},p_{h}-R_{h}p)+ \bigl(u (p_{h}-R_{h}p ),q_{h}\bigr) \\ &\quad=-\bigl(\partial_{t} (p- R_{h}p ),q_{h}\bigr)+ \bigl(u (p-R_{h}p ),q_{h}\bigr)+\bigl(p_{h} (u-u_{h} ),q_{h}\bigr)+(y_{h}-R_{h}y,q_{h}), \end{aligned} \end{aligned}$$

5.14 $$\begin{aligned}& p_{h}(T,x)-R_{h}p(T,x)=0, \quad\forall x\in \Omega. \end{aligned}$$

Hence, letting $w_{h}=y_{h}-R_{h}y$ in (5.11), (5.9) follows from (5.11)-(5.12), Hölder’s inequality, Young’s inequality, Gronwall’s inequality, (3.11), and (5.1). Inequality (5.10) can be similarly derived. □

## Numerical experiment

In this section, we present a numerical example to validate our superconvergence results. Let $\Delta t>0$, $N=T/\Delta t\in \mathbb{Z}^{+}$, $t_{n}=n\Delta t$, $n=0,1,\dots,N$. Set $\varphi^{n}=\varphi(x,t_{n})$ and

$$d_{t}\varphi^{n}=\frac{\varphi^{n}-\varphi^{n-1}}{\Delta t},\quad n=1,2,\ldots,N. $$

By using the backward Euler scheme to approximate the time derivative, we introduce the following fully discrete approximation scheme: find $(y_{h}^{n},p_{h}^{n-1},u_{h}^{n})\in W_{h}\times W_{h}\times K_{h}$ such that

6.1 $$\begin{aligned}& \bigl(d_{t}y_{h}^{n},w_{h} \bigr)+a \bigl(y_{h}^{n},w_{h} \bigr)+\bigl(u_{h}^{n}y_{h}^{n},w_{h} \bigr)= \bigl(f^{n},w_{h} \bigr), \quad\forall w_{h}\in W_{h}, n=1,2,\ldots,N, \end{aligned}$$

6.2 $$\begin{aligned}& y_{h}^{0}(x)=y_{0}^{h}(x), \quad\forall x\in\Omega, \end{aligned}$$

6.3 $$\begin{aligned}& \begin{aligned}[b] &{-} \bigl(d_{t}p_{h}^{n},q_{h} \bigr)+a \bigl(q_{h},p_{h}^{n-1} \bigr)+\bigl(u_{h}^{n}p_{h}^{n-1},q_{h} \bigr)\\ &\quad= \bigl(y_{h}^{n}-y_{d}^{n},q_{h} \bigr), \quad\forall q_{h}\in W_{h}, n=N, \ldots,2,1,\end{aligned} \end{aligned}$$

6.4 $$\begin{aligned}& p_{h}^{N}(x)=0, \quad\forall x\in\Omega, \end{aligned}$$

6.5 $$\begin{aligned}& \bigl(u_{h}^{n}-y_{h}^{n}p_{h}^{n-1},v_{h}-u_{h}^{n} \bigr)\geq0 , \quad\forall v_{h}\in K_{h}, n=1,2, \ldots,N. \end{aligned}$$

Let $\Omega=(0,1)\times(0,1)$, $T=1$, $a=0$, $b=0.25$, and $A(x)$ be a unit matrix. The following example is solved numerically by a precondition projection algorithm (see e.g. [1]), where the codes are developed based on AFEPack, which is freely available.

### Example 1

The data are as follows:

$$\begin{aligned} &p(t,x)=\sin(2\pi x_{1})\sin(2\pi x_{2}) (1-t), \\ &y(t,x)=\sin(2\pi x_{1})\sin(2\pi x_{2})t, \\ &u(t,x)=\min\bigl(0.25,\max\bigl(0,y(t,x)p(x,t)\bigr)\bigr), \\ &f(t,x)=y_{t}(t,x)-\operatorname{div}\bigl(A(x) \nabla y(t,x) \bigr)+u(t,x)y(t,x), \\ &y_{d}(t,x)=y(t,x)+p_{t}(t,x)+\operatorname{div}\bigl(A^{*}(x) \nabla p(t,x)\bigr)-p(t,x)y(t,x). \end{aligned}$$

For brevity, we set

$$|\!|\!|\varphi|\!|\!|= \Biggl(\sum _{n=1-l}^{N-l}\Delta t\bigl\Vert \varphi^{n}\bigr\Vert ^{2}_{L^{2}(\Omega)} \Biggr)^{\frac{1}{2}} $$

and

$$|\!|\!|\varphi|\!|\!|_{1}= \Biggl(\sum _{n=1-l}^{N-l} \Delta t\bigl\Vert \varphi^{n}\bigr\Vert ^{2}_{H^{1}(\Omega)} \Biggr)^{\frac{1}{2}}, $$

where $l=0$ for the control *u* and the state *y*, and $l=1$ for the costate *p*. In Table 1, the errors $|\!|\!|Q_{h}u-u_{h}|\!|\!|$, $|\!|\!|R_{h}y-y_{h}|\!|\!|_{1}$, and $|\!|\!|R_{h}p-p_{h}|\!|\!|_{1}$ on a sequence of uniformly refined meshes are listed. It is consistent with our superconvergence results in Section 5.

**Table 1:** **The errors of Example** **1**

**Δ** ***t***	***h***	$\boldsymbol {|\!|\!|Q_{h}u-u_{h}|\!|\!|}$	$\boldsymbol{|\!|\!|R_{h}y-y_{h}|\!|\!|_{1}}$	$\boldsymbol {|\!|\!|R_{h}p-p_{h}|\!|\!|_{1}}$
1/10	1.0*e* − 1	9.94516*e* − 2	2.68512*e* − 2	4.72410*e* − 2
1/30	5.0*e* − 2	3.41740*e* − 2	8.46440*e* − 3	1.47458*e* − 2
1/90	2.5*e* − 2	1.20365*e* − 2	2.78014*e* − 3	4.68123*e* − 3
1/270	1.25*e* − 2	3.58914*e* − 3	8.83804*e* − 4	1.50701*e* − 3

**Figure 1:**
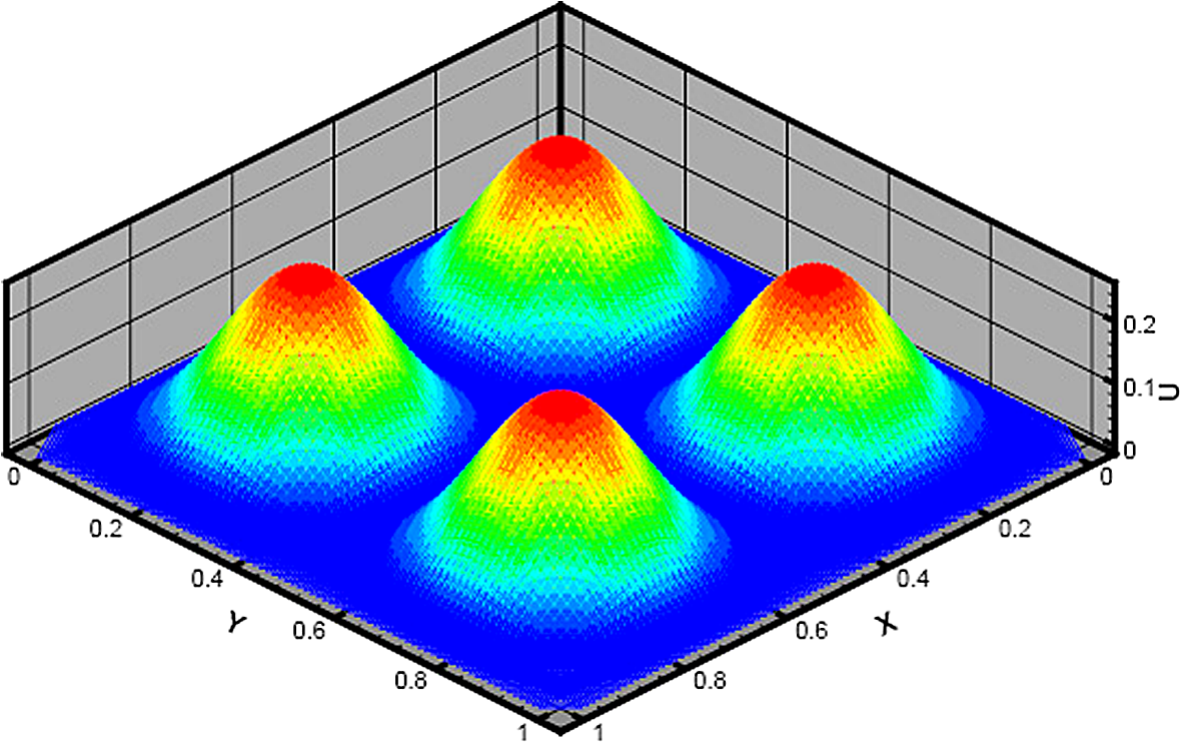
The numerical solution $u_{h}$ at $t=0.5$ in Example 1.

When $h=1.25e{-}2$, $\Delta t=\frac{1}{270}$, and $t=0.5$, we plot the profile of $u_{h}$ in Figure 1.

## Conclusions

Although there has been extensive research on a priori error estimates and superconvergence of finite element methods for various optimal control problems, it mostly focused on linear or semilinear elliptic cases (see, e.g., [2–4, 6]). In recent years, there have been considerable related results for finite element approximation of linear or semilinear parabolic optimal control problems (see, e.g., [9–11]). Although bilinear optimal control problems are frequently met in applications, they are much more difficult to handle in comparison to linear or semilinear cases. There is little work on bilinear optimal control problems. Recently, Yang et al. [5] investigated a priori error estimates and superconvergence of finite element methods for bilinear elliptic optimal control problems. Hence, our results on bilinear parabolic optimal control problems are new.
